# Time-Frequency Aliased Signal Identification Based on Multimodal Feature Fusion

**DOI:** 10.3390/s24082558

**Published:** 2024-04-16

**Authors:** Hailong Zhang, Lichun Li, Hongyi Pan, Weinian Li, Siyao Tian

**Affiliations:** School of Information Engineering, University of Information Engineering, Zhengzhou 450000, China; xinteleixi@sina.com (L.L.); phy98121@163.com (H.P.); lwn916411@163.com (W.L.); tsy-tommy@163.com (S.T.)

**Keywords:** multimodal feature fusion, deep learning, signal recognition, time-frequency diagram, wave-frequency diagram

## Abstract

The identification of multi-source signals with time-frequency aliasing is a complex problem in wideband signal reception. The traditional method of first separation and identification especially fails due to the significant separation error under underdetermined conditions when the degree of time-frequency aliasing is high. The single-mode recognition method does not need to be separated first. However, the single-mode features contain less signal information, making it challenging to identify time-frequency aliasing signals accurately. To solve the above problems, this article proposes a time-frequency aliasing signal recognition method based on multi-mode fusion (TRMM). This method uses the U-Net network to extract pixel-by-pixel features of the time-frequency and wave-frequency images and then performs weighted fusion. The multimodal feature scores are used as the classification basis to realize the recognition of the time-frequency aliasing signals. When the SNR is 0 dB, the recognition rate of the four-signal aliasing model can reach more than 97.3%.

## 1. Introduction

### 1.1. Development Status

With the development of communication technology, the “time and frequency” domain overlap and signal reception, especially wideband signal reception, bring excellent interference. In the complex electromagnetic environment, wide-open receivers often encounter co-channel multi-source signals, that is, in the receiving bandwidth, the same period, there is the existence of multiple communication or non-communication signals [1]. Single-signal identification techniques are more maturely developed, and the traditional method of identifying multi-source aliased signals requires separation followed by identification. It takes a lot of steps and a long time, and the recognition effect is restricted by the separation effect. Especially when the time-frequency aliasing degree of multi-source signals is high, the traditional separation method has a large error under underdetermined conditions, which leads to the failure of the traditional single-signal recognition method. Therefore, exploring a more effective separation method for co-channel time-frequency aliasing signal identification is urgent.

In 2006, Hinton et al., proposed a Deep Belief Network (DBN) and applied it to speech recognition tasks and achieved good results [2]. In 2012, Krizhevsky et al., proposed the concept of a convolutional neural network (CNN), and achieved breakthrough results in image recognition tasks, which laid a foundation for subsequent signal recognition research [3]. In 2016, O’Shea et al., took the lead in applying a CNN to the automatic feature extraction and classification of complex time-domain radio signals [4]. The research team designed a four-layer neural network architecture consisting of two convolutional layers and two fully connected layers, and successfully recognized signals with three analog modulation modes and eight digital modulation modes. Compared with the traditional feature extraction method based on an expert system, this method shows significant performance advantages. The results not only show the high adaptability of a CNN in processing time series data, but also confirm its efficiency and accuracy in automatic feature extraction and classification tasks. With the successful application of a CNN in the field of signal recognition, more and more algorithms have been proposed. Ref. [5] compare the performance of Long Short-Term Memory (LSTM) and a CNN in radio signal modulation recognition tasks in detail. The simulation results show that the recognition rate of the neural network to the signal is not affected by the depth of the network and the size of the filter, thus revealing the flexibility and robustness of the network structure selection in the field of modulation recognition. Ref. [6] proposed a classification algorithm based on a transformer and denoising autoencoder (DAE). The algorithm combines the denoising autoencoder component in the DAE_LSTM model and the Residual Stack design in the Res-Net architecture, and finally integrates the attention mechanism of the transformer to enhance the feature extraction and sequence modeling capabilities. The experimental results show that the proposed algorithm performs well on the public dataset RadioML2018.01A. Ref. [7] proposed a multimodal attention mechanism signal modulation recognition method based on Generative Adversarial Networks (GANs), a CNN, and LSTM to solve the problem of the low recognition accuracy of spread spectrum signals under low signal-to-noise (SNR) conditions. In this method, the GAN is used to denoise the time-frequency image, and then the time-frequency image and I/Q data are input into the recognition model based on a CNN and LSTM, and the attention mechanism is added to the model to realize the high-precision recognition of ten kinds of signals such as MASK and MFSK.

To sum up, time-frequency aliasing signal separation based on machine learning has become a research hotspot [8], mainly divided into two identification methods based on decision trees and neural networks. In the decision tree-based recognition method, Ref. [9] extract eight kinds of features to identify twelve kinds of signals, and the feature selection is complicated. In the neural network-based recognition method, Ref. [10] extracts instantaneous features and higher-order cumulative volume features and uses the BP network for the intra-class recognition of phase-shift keying (PSK) and quadrature amplitude modulation (QAM) signals, but the complexity of the algorithm is high. Ref. [11] dataset’s SNR is fixed at 4 dB and 10 dB, and Ref. [12] does not investigate mixed signals composed of source signals with different code rates; all of them have the problem of poor dataset generalization ability. Refs. [13,14] use the Deep Convolutional Neural Network (DCNN) network and Seg-Net network to extract time-frequency graph features to achieve signal separation and identification, respectively, but the features are selected singly, and the intra-class identification of modulated signals cannot be achieved.

Currently, machine learning-based signal recognition methods are ineffective for intra-class signals in practical applications, mainly because intra-class signals are challenging to recognize due to the same modulation of the broad classes and similar single-dimensional features. To solve this problem, this article proposes a time-frequency aliasing signal recognition method based on multi-mode fusion (TRMM); the method first performs multidimensional feature extraction from two modes, a time-frequency diagram and wave-frequency diagram, and then establishes a pixel-level weighted fusion decision-maker to adjudicate each pixel; the method achieves inter-class recognition as well as satisfactory intra-class recognition. At an SNR of 0 dB, the recognition rate of the four-signal aliasing model can reach more than 97.3%.

### 1.2. Organization

This article is organized as follows: Section 2 introduces the model of the time-frequency aliasing signals and the evaluation criteria of the recognition performance; Section 3 describes in detail the preprocessing method of the time-frequency aliasing signals; Section 4 discusses the neural network and fusion strategy; Section 5 gives the simulation results and performs the performance analysis; and Section 6 concludes the article.

## 2. Signal Model

### 2.1. Mixed Signal Model

**Figure 1:**
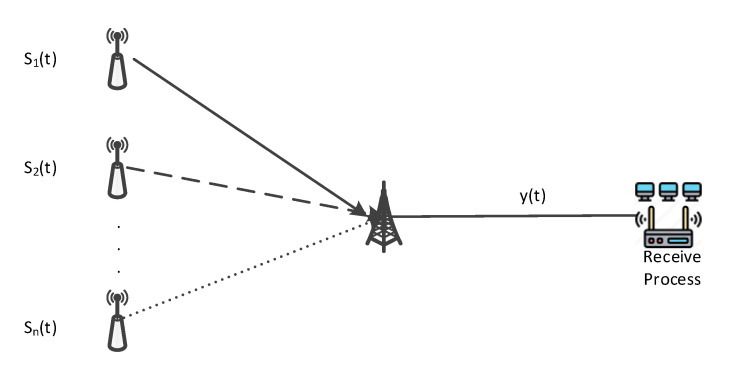
Signal mixing model.

The linear transient mixing model is shown in Figure 1:

Where m is the number of receiving channels and n is the number of source signals. Expression of the model as Equation:

(1) $$x\left(t\right)=\sum_{i=1}^{n}A_{i}s_{i}\left(t\right)+\nu (t),$$

where $A_{i}$ is the amplitude of each original signal component $s_{i}\left(t\right)$, and $v\left(t\right)$ is the additive Gaussian white noise.

In the real communication environment, the signal received by the receiver contains not only the modulation-type signal in the general communication business channel, but also various radar signals. To be close to reality, $s_{i}\left(t\right)$ in this article refers to nine mainstream communication signals and two common radar signals, including amplitude modulation (AM), binary amplitude-shift keying (2ASK), binary frequency-shift keying (2FSK), quaternary frequency-shift keying (4FSK), binary phase-shift keying (BPSK), differential quadrature reference phase-shift keying (DQPSK), 8 phase-shift keying (8PSK), 16-ary quadrature amplitude modulation (16QAM), 32-ary quadrature amplitude modulation (32QAM), linear frequency modulation (LFM), and even quadratic frequency modulation (EQFM) [9]. As a single-signal set, the time-frequency aliasing signal is generated.

### 2.2. Frequency-Domain Analysis

The different frequency distributions of the components in the aliased signals lead to different mixing degrees of the aliased signals. In this article, one signal $S_{i}$ is selected from the above eleven signal models and mixed with several other signals within a time interval.

The time-domain aliasing degree $M_{t}$ is defined as

(2) $$M_{t}=\frac{t_{\;\;S_{i}}^{mix}}{t_{S_{i}}^{exist}}$$

where $t_{\;\;S_{i}}^{mix}$ denotes the time the signal $S_{i}$ is aliased with other signals, and $t_{S_{i}}^{exist}$ denotes the time the signal $S_{i}$ is present. In our experiment, the default time-domain aliasing degree $M_{t}=100\%$.

The frequency domain aliasing degree $M_{f}$ is defined as

(3) $$M_{f}=\frac{f_{\;\;S_{i}}^{mix}}{f_{S_{i}}^{exist}}$$

wherein $f_{\;\;S_{i}}^{mix}$ denotes the bandwidth of the signal $S_{i}$ overlapped with other signals, and $t_{S_{i}}^{exist}$ denotes the bandwidth of the signal $S_{i}$. In particular, $S_{i}$ denotes the signal with the narrowest bandwidth in the frequency domain when the component in the aliased signal is greater than two.

### 2.3. Evaluation Criteria

In this article, the single-signal recognition accuracy $P_{r}$ is defined as

(4) $$P_{r}=\frac{N_{r}}{N_{s}}\times 100\%$$

where $N_{r}$ denotes the number of signals accurately recognized by the algorithm and $N_{s}$ denotes the total number of test signals.

In this article, the aliasing signal recognition accuracy $P_{m}$ is defined as

(5) $$\begin{array}{cc} P_{m}=\frac{1}{I}\sum_{i=1}^{I}\left(\frac{N_{r}^{i}}{N_{s}^{m}}\times 100\%\right), & I=\left\{\begin{array}{ll} 2 & \mathrm{Dual}-\mathrm{signal}\\ 3 & \mathrm{Three}-\mathrm{signal}\\ 4 & \mathrm{Four}-\mathrm{signal} \end{array}\right. \end{array}$$

where $N_{s}^{m}$ denotes the total number of aliased signals tested, and $N_{r}^{i}$ denotes the total number of class i component signals accurately identified by the algorithm.

In this article, the average recognition rate $P_{a}$ is defined as

(6) $$\begin{array}{cc} P_{a}=\frac{1}{J}\sum_{j=1}^{J}\left(\frac{N_{r}^{j}}{N_{s}^{j}}\times 100\%\right), & J=\left\{\begin{array}{ll} 11 & \sin\mathrm{gle}\;\mathrm{signal}\\ 10 & \mathrm{dual}-\mathrm{signal}\\ 4 & \mathrm{Three}-\mathrm{signal}\\ 6 & \mathrm{Four}-\mathrm{signal} \end{array}\right. \end{array}$$

where $N_{r}^{j}$ denotes the number of class j signals or aliasing models accurately identified, and $N_{s}^{j}$ denotes the total number of class j signals or aliasing models tested.

## 3. Multimodal Data Construction

In the field of time-frequency aliasing signal processing, a single-modal feature is usually selected, ignoring the complementarity between different feature modes of the signal [15]. By correlating the signal’s homologous and heterogeneous features, drawing on the advantages of different modal features, the effective integration of modal information can be accomplished, and the feature expression ability can be improved.

### 3.1. Time-Frequency Diagram

In practical applications, the communication signals received by the receiver are non-smooth signals with the performance of overlapping in the time domain, aliasing in the frequency domain, and poor sparsity, and the time-frequency domain analysis expresses the non-smooth signals as a two-dimensional function of the frequency and time, which better reveals the time-frequency dynamics of the signals and non-smooth characteristics. Therefore, this article transforms the signal to the time-frequency (TF) domain for analysis. Time-frequency analysis methods are divided into linear time-frequency analysis and quadratic time-frequency analysis, and typical linear time-frequency analysis includes short-time Fourier transform (STFT), wavelet transform (WT), etc. STFT, as a linear transformation, does not generate cross-interference terms and has a strong processing capability and resistance to frequency-domain diversity signals. It has a strong processing ability and anti-interference ability. In this article, STFT is selected as a means of time-frequency analysis.

The STFT transform equation of the signal $S\left(t\right)$ is expressed as

(7) $$STFT_{S}\left(t,f\right)=\int_{-\infty }^{\infty }\left[S\left(u\right)g^{*}\left(u-t\right)\right]e^{-j2\pi fu}du$$

where * stands for the complex conjugate and $g\left(t\right)$ is the window function.

**Figure 2:**
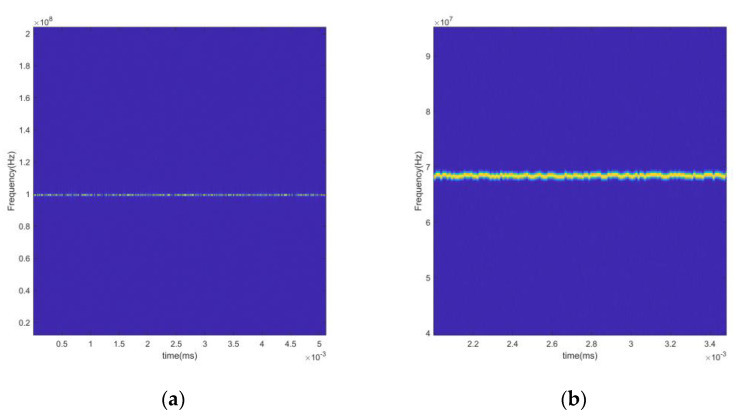

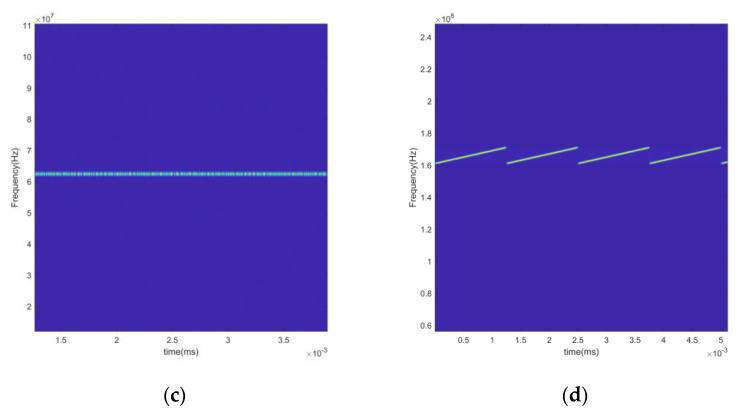
Example of time-frequency diagrams for different signals ((**a**): 2ASK; (**b**): 4FSK; (**c**): 8PSK; (**d**): EQFM).

The time-frequency diagram is a visual presentation of the magnitude values of the STFT transform results, which more accurately reveals the signals’ transient characteristics and frequency dynamics. The time-frequency diagrams of the 2ASK, 4FSK, 8PSK, and LFM signals are given in Figure 2. The sampling rate is 512 MHz, and the symbol rate is 10–200 kHz. Considering the bandwidth and the actual rendering effect, the number of sampling points is set to 1535. The 2ASK signal uses “OOK” modulation, sending “0” corresponds to no energy in the graph, and sending “1” corresponds to the energy in the graph. The 4FSK signal has four frequency variations in the time-frequency domain. The 8PSK signal has no frequency change; the LFM signal has a linear slope.

### 3.2. Wave-Frequency Diagram

Signal waveform refers to the expression form of the signal in the time domain or space domain, that is, the graph of the signal changing with the time or the shape of the spatial distribution. It depicts how the amplitude, frequency, phase, and other characteristics of the signal change with time.

**Figure 3:**
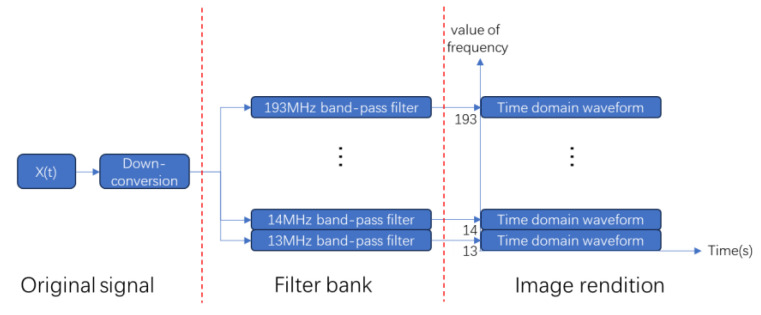
Flowchart for generating wave-frequency diagrams.

When the signal is processed by STFT, the resolution of the frequency domain is improved, but the time resolution is reduced when the window function is longer. On the contrary, when the window function is short, the temporal resolution increases, but the frequency-domain resolution decreases. In practical applications, it is usually necessary to make a compromise between the time and frequency resolution, and a part of the spectral resolution or time resolution will be lost, resulting in information loss. Therefore, this article introduces the concept of a wave-frequency diagram. The wave-frequency diagram is a waveform diagram that moves the waveform information to the position corresponding to the signal’s carrier frequency after the down-conversion of the signal and contains the complete time-domain characteristics and carrier frequency information; within the frequency range of 13–193 MHz, a bandpass filter is set every 1 MHz to filter the signal, and the time-domain waveform of the filtered signal is placed on the corresponding vertical axis to form the wave-frequency diagram. Figure 3 shows the flow of the wave-frequency diagrams’ generation.

**Figure 4:**
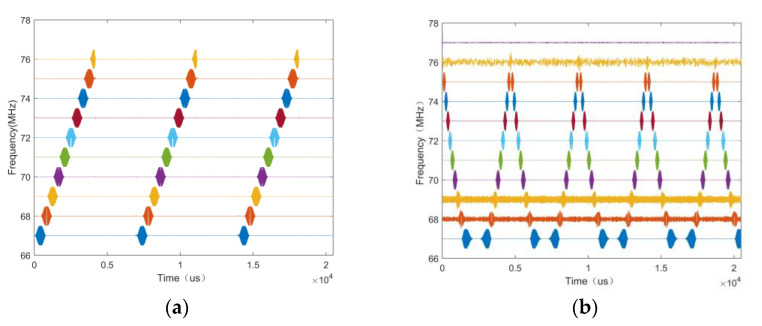
Wave-frequency diagrams of different signals ((**a**): LFM; (**b**): EQFM + BPSK + DQPSK + 8PSK).

The wave-frequency diagrams of the LFM and EQFM + BPSK + DQPSK + 8PSK signals are given in Figure 4, and it can be seen that the wave-frequency diagrams of the LFM and EQFM signals exhibit apparent time-domain features.

### 3.3. Image Preprocessing

**Figure 5:**
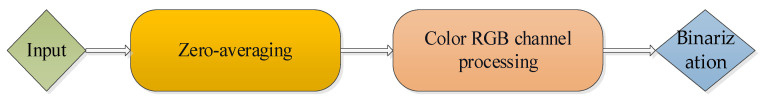
Flow chart of image preprocessing.

The binarization preprocessing of the time-frequency and wave-frequency diagrams can strengthen the feature contrast in the image and remove the color interference and the influence of channel noise; at the same time, it can reduce the image dimension, reduce the amount of computation of the neural network in the forward propagation, accelerate the inference process, and improve the operation speed. The preprocessing process is shown in Figure 5.

**Figure 6:**
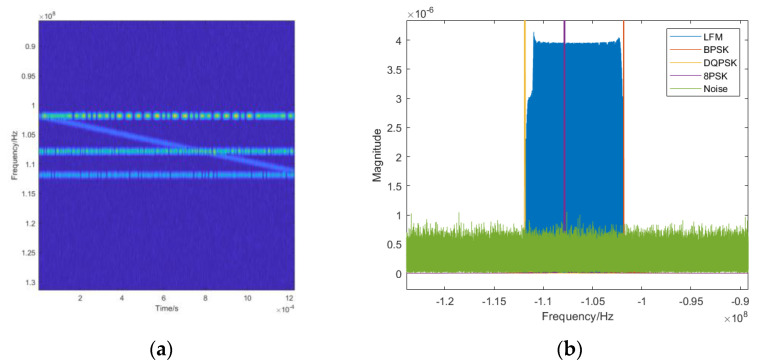
Time-frequency diagram and corresponding spectrum of BPSK + DQPSK + 8PSK + LFM model ((**a**). time-frequency distribution, (**b**). spectrum waveform).

Taking the time-frequency diagram as an example, the steps of the image preprocessing are explained as follows:

Input: The time-frequency diagram of the time-frequency aliased signal with noise is taken as the input, as shown in Figure 6.

Zero-averaging: Each pixel value in the time-frequency diagram is subtracted from the average value of the row in which the pixel is located so that the pixel value varies between positive and negative, which helps to improve the learning efficiency, performance, and generalization ability of the neural network, as well as to simplify the computation process and reduce potential numerical problems.

Color RGB channel processing: As shown in Figure 6, in the actual channel environment, the signal is surrounded by noise, which results in some degree of distortion. By observing the pixel points of the signal and the noise, it can be seen that at the signal’s aliasing, the noise background is mainly composed of pixel points on the B channel, while the main pixel points of the signal are concentrated in the G channel. At this point, all pixel points within the R and B channels are discarded and sharpened to enhance the signal characteristics.

**Figure 7:**
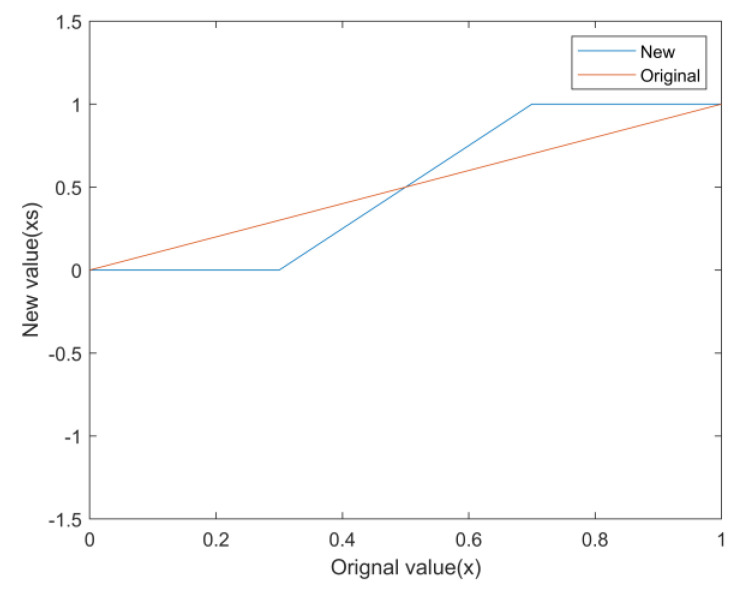
Sharpening processing schematic.

The sharpening process can be expressed as

(8) $$x_{s}=\left\{\begin{array}{cc} y_{1} & x<x_{1}\\ \frac{y_{2}-y_{1}}{x_{2}-x_{1}}\times \left(x-x_{1}\right) & x_{1}\leq x\leq x_{2}\\ y_{2} & x>x_{2} \end{array}\right.$$

where $\left[x_{1},x_{2}\right]$ denotes the value range of the original pixel point, $\left[y_{1},y_{2}\right]$ denotes the value range after expansion, x is the value of the original pixel point, and $x_{s}$ is the pixel value after sharpening. When $\left[x_{1},x_{2}\right]=\left[0.3,0.7\right]$, $\left[y_{1},y_{2}\right]=\left[0,1\right]$, the pixel value change curve is shown in Figure 7.

Binary processing: After sharpening the image, binary processing is performed. After sharpening the image, the difference in the original image pixel value becomes larger, the signal color is more prominent, the binarization threshold is more reasonable, and the binarization effect is better. The binarization threshold can be determined by the following equation [16]:

(9) $$\sigma_{B}^{2}\left(k\right)=P_{1}\left(k\right)\cdot {\left(m-m_{1}\left(k\right)\right)}^{2}+P_{2}\left(k\right)\cdot {\left(m_{2}\left(k\right)-m\right)}^{2}$$

where $\sigma_{B}^{2}\left(k\right)$ is the between-class variance at threshold k, $P_{1}\left(k\right)$ and $P_{2}\left(k\right)$ are the probabilities of the foreground and background, respectively, m is the overall average gray value, and $m_{1}\left(k\right)$ and $m_{2}\left(k\right)$ are the average gray value of the foreground and background, respectively.

**Figure 8:**
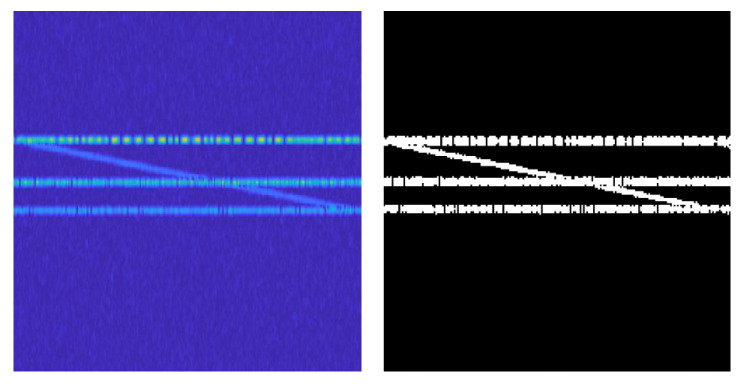
Comparison of image preprocessing effect.

The effect of the image preprocessing is shown in Figure 8. By preprocessing the original time-frequency image, the noise interference is successfully eliminated from the background while accurately retaining the key features of the signal, and the denoising effect is good.

## 4. Multimodal Deep Learning-Based Signal Recognition Methods

**Figure 9:**
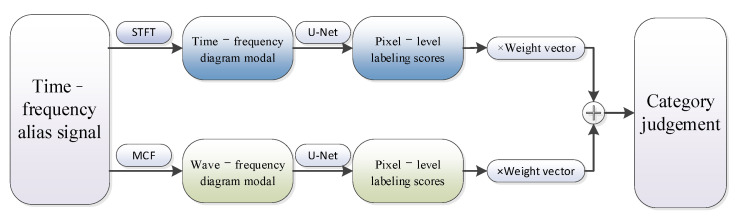
Basic flowchart of TRMM method processing.

**Figure 10:**
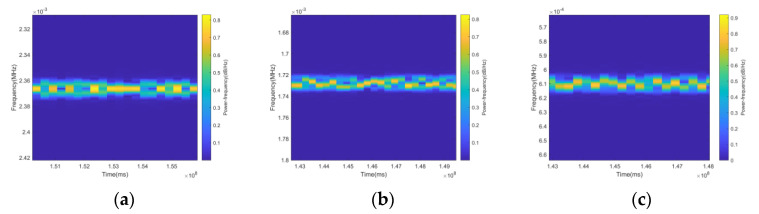
Time-frequency diagram of MPSK signal ((**a**): BPSK; (**b**): QDPSK; (**c**): 8PSK).

A single-modal feature (SMF) cannot effectively identify the modulation mode of the signal within the class, so the proposed TRMM method extracts the time-frequency domain features and wave-frequency domain features pixel by pixel. Then, it performs the weighted fusion to realize the signal identification by using the multimodal feature scores as the classification basis. The basic flow is shown in Figure 9:

The signals within the class use the same modulation method as the broad class; only the number of modulation progressions is different, and the time-frequency domain characteristics are difficult to distinguish. The time-frequency diagram of the MPSK (M = 2, 4, 8) signals is given in Figure 10, and it can be seen that these three signals have similar characteristics in the time-frequency domain, which makes it difficult to distinguish between them.

**Figure 11:**
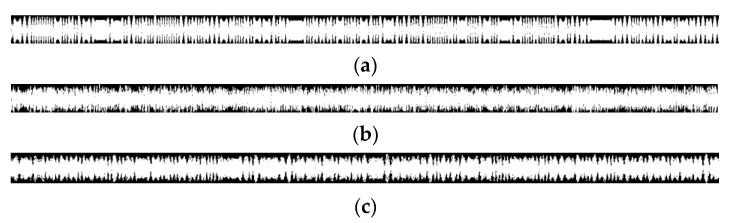
Wave-frequency diagram of MPSK signal ((**a**): BPSK; (**b**): QPSK; (**c**): 8PSK).

The signals are highly feature-dense in the time domain and contain rich raw information but are challenging to distinguish with the naked eye; the neural network can self-feed, learn, and extract critical features in the time domain, capturing the nonlinear relationships and enabling pixel-level classification. An example of a wave-frequency diagram of an MPSK (M = 2, 4, 8) signal is given in Figure 11.

### 4.1. Pixel-Weighted Averaging

In general, the training process of semantic segmentation networks requires that the distribution of pixels in each category in the sample set achieves an elemental equilibrium. However, due to the inherent limitation of the time-frequency characteristic pattern of the signal, the pixels of different categories cannot be uniformly distributed throughout the time-frequency domain, resulting in many pixels being discriminated as background labels. If adopted directly during training, this unbalanced label distribution will cause the network to bias the signal categories that account for more pixels during the learning process. In order to compensate for this imbalance and optimize the learning process, this article employs the Inverse Probability Weighting (IPT) method [17] to assign appropriate weights to each signal category.

(10) $$Pro^{S_{i}}=\frac{N^{S_{i}}}{N_{SUM}^{S_{i}}}$$

where $Pro^{S_{i}}$ denotes the probability of the signal class, $N^{S_{i}}$ denotes the number of signal pixels of class $S_{i}$ in the training set, $N_{SUM}^{S_{i}}$ denotes the total number of pixels containing the labeled images of class $S_{i}$, i denotes the signal class, and in this article, i=1,2,⋯,12. The pixel weighting is then denoted as

(11) $$\omega_{S_{i}}=\frac{mid\left[Pro^{\cup S_{i}}\right]}{Pro^{S_{i}}}$$

where $mid\left[•\right]$ denotes taking the median of the array, and ∪• denotes forming an array from the numbers.

**Figure 12:**
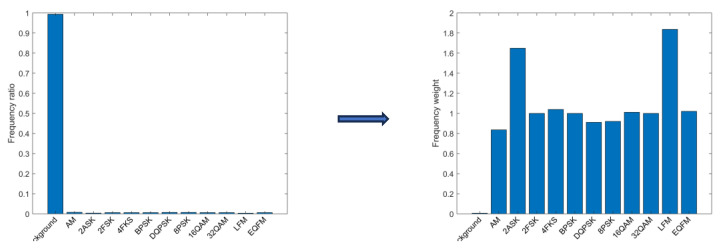
Comparison of pixel frequencies and category weights for each type of signal.

When dealing with the class imbalance problem, by calculating and applying the weights of each class, the samples of different classes can be given different degrees of importance in the model training phase, as shown in Figure 12. Specifically, when the class weights are obtained, these weights are introduced into the training process of the network as parameters, which can effectively reduce the learning bias and performance degradation caused by class imbalance [18].

### 4.2. Feature Extraction Based on U-Net Networks

**Figure 13:**
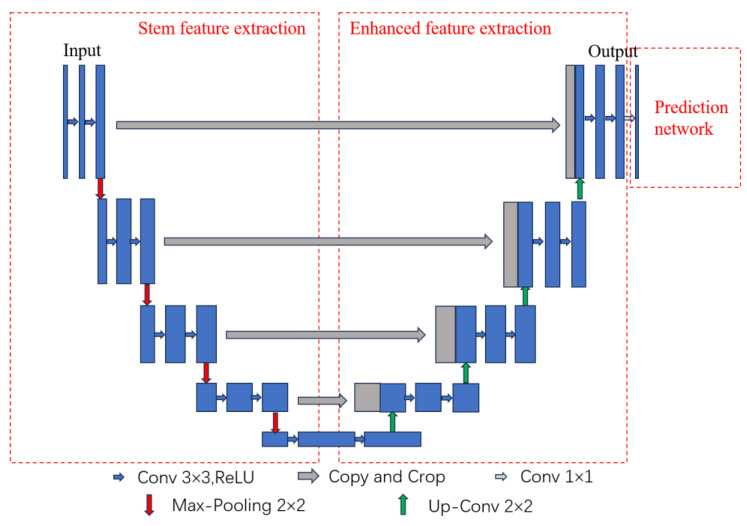
Structure of the U-Net network.

The U-Net network is a symmetric U-shape structure, a pixel-level semantic segmentation model. The central feature extraction part uses convolution and pooling for dimensionality reduction to increase the image channels and obtain low-dimensional feature information; the enhanced feature extraction part uses multi-scale feature fusion and other methods to repair feature details, restore image dimensions, and include feature information. The network structure is shown in Figure 13 [19].

The figure shows that the U-Net network is divided into five layers. The blue arrows indicate 3 × 3 convolution for feature extraction, and a Bn layer is added between ReLU and convolution without changing the width and height of the feature layer. It allows the characteristic pattern to be spliced directly without center cropping. Red arrows indicate 2 × 2 max pooling, which downscales and compresses the characteristic pattern by 2 × 2 filters. Gray arrows indicate feature fusion, i.e., the features extracted after convolution and pooling in each layer are linked to the corresponding upsampling layer, which ensures that the network obtains global and local information at different layers and improves the accuracy of image segmentation. The green arrow indicates upsampling, where the image is deconvolved to recover the dimensionality, giving the image a higher resolution and recovering some of the image features.

The U-Net network enables pixel-level classification, and the network outputs the category of each pixel point. As shown in Figure 13, the encoded matrix is upsampled on the right side, the size of the matrix becomes more extensive, and it is superimposed with the matrix of the same size on the left side in the channel direction (grey arrows). After several superimpositions, the matrices are mapped to probability values to classify at the pixel level. As the network layers deepen, the resulting characteristic patterns have a larger field of view, allowing a more precise determination of the signal class to which the pixel point belongs. Therefore, the U-Net network is chosen to extract the features of the signal on a pixel-by-pixel basis, which allows the extraction of high-precision features of the signal.

#### 4.2.1. Trunk Feature Extraction

**Figure 14:**
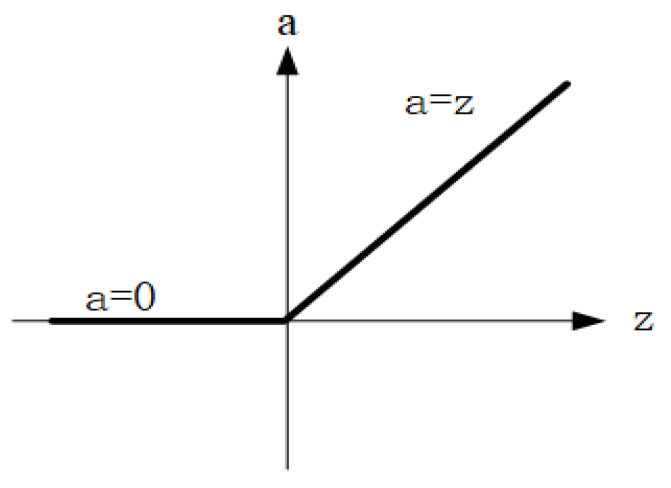
The image of the ReLU function.

The backbone feature extraction structure consists of convolutional and maximum pooling layers. In the convolutional layer, the input data will be nonlinearly transformed and linearly transformed by the activation function (ReLU) and the weight matrix and then stacked with the maximum pooling layer for feature extraction to obtain the initial effective feature layer. The expression of the ReLU function is, and its image is shown in Figure 14.

**Figure 15:**
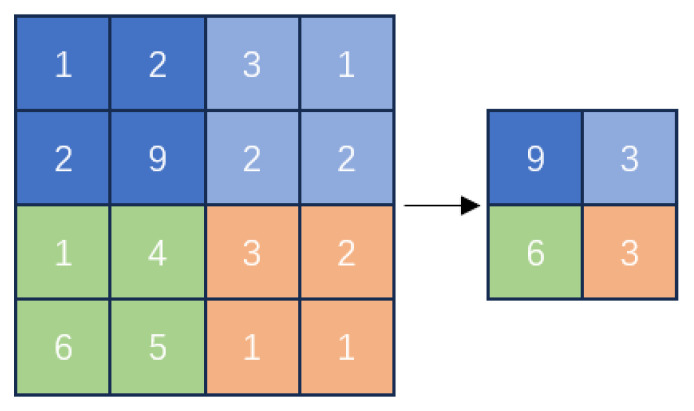
Schematic of maximum pooling.

Maximum pooling has a pooling kernel size of 2, as shown in Figure 15:

When the time-frequency image is input into the U-Net network for trunk feature extraction, the image needs to be processed with pixel-weighted averaging, and the steps are as follows:

1. The total number of time-frequency image pixels for one round of training of the statistical network is m.
2. Calculate the number of effective pixels for each category of signals as $m_{s}^{i}=\omega_{S_{i}}\times m_{s}$ according to the corresponding pixel-weighting weights.
3. Each category signal multiplies the category score of the corresponding $m_{s}^{i}$ effective pixels by the loss function and puts them into the next round of training.

In forward propagation, the neural network calculates the loss function by comparing the predicted result and the actual label. The loss function measures the error between the expected and actual results, providing a basis for subsequent weight adjustment. The expression of the loss function is

(12) $$Loss=\frac{1}{N}\sum_{i}L_{i}=-\frac{1}{N}\sum_{i}^{N}\sum_{c=1}^{M}y_{ic}\ln(p_{ic})$$

where N is the number of samples; M is the number of categories; $y_{ic}$ is a sign function that takes one if the proper category of sample i is c and 0 otherwise; and $p_{ic}$ is the predicted probability that the observed sample i belongs to category c.

#### 4.2.2. Enhanced Feature Extraction

The enhancement feature extraction structure, also known as the expansion path, is designed to map the high-level features generated by the backbone feature extraction structure back to the original image size and consists of an upsampling operation and a convolutional layer. Each step of the enhanced feature extraction structure is mirrored with the corresponding step of the backbone feature extraction structure to form a jump connection, which connects the shallow features in the backbone feature extraction structure directly to the corresponding level of the enhanced feature extraction structure, and at the same time compensates for the loss of positional information that the pooling layer may cause. This connection mechanism allows the network to utilize local features and global context information, resulting in accurate predictions without sacrificing spatial resolution.

#### 4.2.3. Extraction Method

After the time-frequency diagram and wave-frequency diagram images are preprocessed, the target features in the image are more prominent, which is convenient for contour extraction and shape analysis. The time-frequency diagram is sent to the U-Net network for pixel-by-pixel category judgment, obtaining the category judgment for each pixel and obtaining the confidence level of the category; for the overlapping signals, the overlapping area is marked as “overlapping category” for subsequent processing. The wave-frequency diagram is fed into the U-Net network for segmentation $\left(768/20480,64\right)$, the category judgment is made region by region, the categories of all the pixels in the segmented region are counted, and the category to which the most pixels belong is taken as the category of the segmented region of the wave-frequency diagram. The discriminative score of the category is obtained.

#### 4.2.4. Split Output

**Figure 16:**
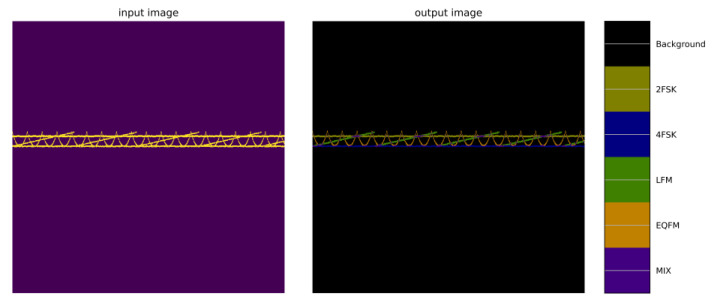
Graph of the U-Net network segmentation output.

The U-net network is often used for image segmentation tasks. Unlike traditional convolutional neural networks for classification tasks, its output is not a class label for the entire image, but a classification for each pixel in the image, and the class of each pixel is represented by a different color. In this chapter, the signal and background in Section 2.1 are divided into twelve categories, and each pixel is segmented by the U-Net network and its category is identified, and its category label and the confidence of this category are output. The twelve categories are represented by different colors, as shown in Figure 16. This representation method makes the final segmentation result intuitive and easy to understand. The distribution and boundaries of different categories of regions can be clearly seen.

### 4.3. Inductor Setup

The inference process is when a neural network with parameters already determined in training performs operations to predict or infer new input data. Unlike the forward propagation used in the training process, the inference process does not need to compute a loss function and perform a back-propagation algorithm. The weights and bias values in the inference process are fixed and are not updated again [11].

The modal fusion weights of the wave-frequency and time-frequency diagrams can be expressed as

(13) $$P_{i}={\vec{w}}_{wi}\times P_{wi}+{\vec{w}}_{si}\times P_{si}$$

where $P_{i}$ is the probability that the signal is judged as category i, ${\vec{w}}_{wi}$, ${\vec{w}}_{si}$ are the weights of the wave-frequency and time-frequency diagrams of the signal i, $P_{wi}$, $P_{si}$ is the probability that the signal is judged as category i by the time-frequency and wave-frequency diagrams alone. The time-frequency diagram is discriminated pixel by pixel in the network, and each pixel receives a category score $P_{si}$, and a weighted score is output as ${\vec{w}}_{si}⊙P_{si}$. Assuming that pixel A corresponds to the moment t and frequency f in the time-frequency diagram, the waveform region in the wave-frequency diagram of this pixel (non-background pixel) corresponding to the frequency f is J. After segmentation of the wave-frequency diagram, the category judgment is carried out in the network. The category score of region J is obtained as $P_{wi}$, and the weighted score of region J is output as ${\vec{w}}_{wi}⊙P_{wi}$. The final score obtained from the weighted score of pixel A and the weighted score of its corresponding region J in the wave-frequency diagram is the discrimination score $P_{i}$ of pixel A.

After the U-Net network adjudicates the time-frequency and wave-frequency diagrams separately, the respective adjudication results may need to be revised due to the limitation of unimodal features. In order to improve the final recognition rate, it is necessary to weigh the fusion of the time-frequency and wave-frequency diagrams and set different weights to make the fused judgment results reach the optimum. 2FSK and 4FSK show two and four states in the time-frequency domain, respectively, which have more significant differentiation ability than the wave-frequency modes and need to be given higher weights in the time-frequency domain; DQPSK has the same number of states in the time-frequency domain compared to 4FSK, and its wave-frequency modes have more significant differentiation ability compared to the wave-frequency modes. Compared with 4FSK, DQPSK has the same number of states in the time-frequency domain, and its wave-frequency domain modes have more significant differentiation ability and need to be given higher weighting in the wave-frequency domain; 16QAM compared with 32QAM, the number of states are fewer in the time-frequency domain, have more significant differentiation ability, and need to be given higher weighting in the time-frequency domain, then the 32QAM in the wave-frequency domain needs to give a higher weighting; LFM and EQFM in the time-frequency domain have a significant differentiation ability, need to be given in the time-frequency domain capability, and need to be given a higher weighting in the time-frequency domain. Under the condition of randomly setting the SNR (0–20 dB), 100 single signals of each of the 11 types are generated, and the recognition test is carried out only through the time-frequency diagram modes or only through the wave-frequency diagram modes, and the recognition rate of each single signal is shown in Table 1. Combining the recognition rate statistics of each modality and the above analysis, the following weights are given (Table 2):

### 4.4. Threshold Filtering

After the time-frequency and wave-frequency diagrams of the aliased signals are fed into the trained neural network, the output result is an image of the same size as the input image, which also contains the information on the classification labels. Usually, semantic segmentation is completed at this stage, while in the modulation recognition of the aliased signal, individual pixel labeling errors may lead to modulation recognition errors. Therefore, to further improve the final signal recognition rate, this article proposes introducing a threshold filter after the output of the U-network [12]. The neural network obtains the discriminative score of the pixel category of an aliased signal when obtaining the category label of the pixel. At this point, a high threshold is set to filter the pixel categories, retaining the signal categories with high scores and removing those with low scores. Even if there are some pixels with inaccurate category labels, the modulation type of each component of the aliased signal can be accurately identified by applying the network’s subsequent threshold filter.

## 5. Simulation Experiments

### 5.1. Dataset

In order to verify the robustness of the algorithm, the modulation parameters of the signals are set to be randomly generated within the expected range: the code rate is 10–200 kHz, the SNR is 0–20 dB, the frequency is 13–193 MHz, and the bandwidth of the radar signal sweep is 10 MHz. A total of 1000 each of 11 single-signal models in the training set are generated; 300 each of 19 two-signal aliasing models and 5 three-signal aliasing models are generated, and the degree of aliasing is randomly generated within the range of 25–100%. The tests focused on generating 100 signals of each type in each parameter condition for eleven single-signal, ten two-signal aliasing models, four three-signal aliasing models, and 6 four-signal aliasing models (Table 3), with an SNR ranging from 0 to 20 dB in 4 dB steps, and aliasing degrees of 0.25, 0.5, 0.75, and 1. Four-signal aliasing models included the aliasing of both inter- and intra-class signals. The time-frequency pixel size of all the signals was set to 768 × 768 uniformly, and the wave-frequency pixel size was set to 2048 × 64.

Once the dataset is created, it is necessary to label the images. The time-frequency map of each component signal is labeled pixel by pixel and mapped to the time-frequency map of the mixed signal. The pixels with overlapping component signals are uniformly marked as “mix” and output as aliasing pixel labels.

### 5.2. Status of Network Training

**Figure 17:**
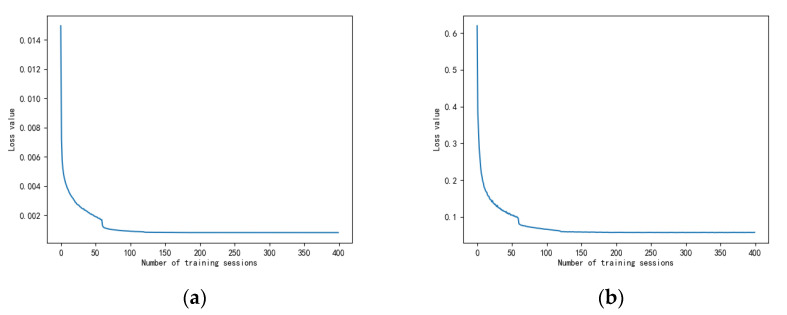
Trends in loss values for U-Net networks ((**a**). trend of time-frequency plot loss values with training rounds; (**b**). trend of wave-frequency plot loss values with training rounds).

The initial learning rate of the network was set to 0.001, and 400 rounds of training were performed by the computer. The left graph in Figure 17 shows the trend of the loss values with the training rounds under the time-frequency plot dataset, and the exemplary chart indicates the direction of transformation of the loss values with the training rounds under the wave-frequency plot dataset. The time-frequency plot has a loss value of 0.0150 at the beginning of round 0, and the loss value drops to 0.0072 at the end of the round. When Epoch = 244, the loss value is 0.0008, stabilizes, and reaches its minimum. The loss value of the wave-frequency plot is 0.6193 at the beginning of round 0, and at the end, the loss value drops to 0.4135. When Epoch = 323, the loss value is 0.0573, levels off, and reaches its minimum. Therefore, the time-frequency domain network parameters are selected as Epoch = 244, and the wave-frequency diagram network parameters are chosen as Epoch = 323.

### 5.3. Analysis of Results

#### 5.3.1. Analysis of Single-Signal Recognition Results

For the purpose of contrasting the efficacy of single-modal recognition with that of multimodal recognition, this study conducted experiments on single signals at an SNR of 0 dB, 4 dB, 8 dB, 12 dB, 16 dB, and 20 dB. A corpus of 100 distinct single signals was generated for each SNR level to evaluate the time-frequency pattern recognition and the fusion mode recognition, respectively. The findings of these evaluations are documented in Table 4. The data elucidated in Table 4 reveal that, under the single-modal feature set, the DQPSK signal exhibited the lowest recognition rate when the SNR was at 0 dB, standing at a mere 16%. This reduced performance is attributed to the propensity for its time-frequency domain characteristics to be conflated with those of the BPSK, 8PSK, and 16QAM signals; however, the time-frequency attributes of the other signals were sufficiently distinct, allowing for their accurate identification. It is evident from the results that the recognition accuracy for single signals progressively enhances as the SNR escalates. Notably, upon reaching an SNR of 12 dB, the recognition accuracy for the test set of the single signals devised in this study attained a perfect score of 100%. In the multimodal feature space, the recognition rate for the DQPSK signal at an SNR of 0 dB was recorded at 99%, which constitutes an 83% improvement over the single-modal test outcomes. In an overall assessment, the average recognition rate for single signals was measured at 81.36% within the single-modal feature context, in contrast to the 99.81% achieved under the multimodal feature framework. The juxtaposition of these methodologies unequivocally demonstrates that the multimodal fusion strategy significantly augments the capability to recognize individual signals.

#### 5.3.2. Analysis of Dual-Signal Aliasing Model Identification Results

**Figure 18:**
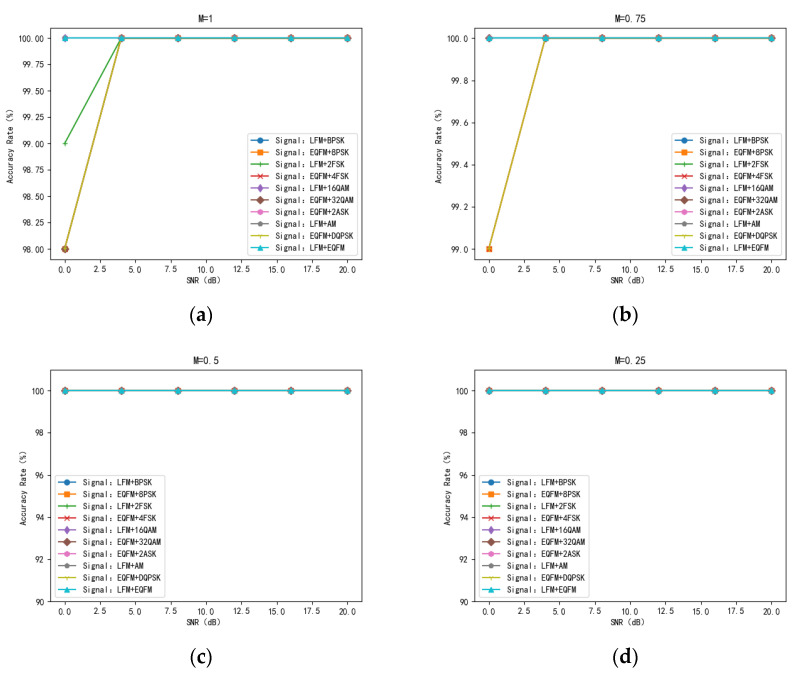
Trend of recognition accuracy of dual signals in multimodal mode with SNR at different aliasing degrees ((**a**). trend of recognition accuracy versus SNR for dual signals in multimodal mode with 25% overlap; (**b**). the trend of recognition accuracy versus SNR for dual signals in multimodal mode with 50% overlap; (**c**). the trend of recognition accuracy versus SNR for dual signals in multimodal mode with 75% overlap; (**d**). the trend of recognition accuracy versus SNR for dual signals in multimodal mode with 100% overlap).

In order to verify the recognition effect of the TRMM method in the dual-signal time-frequency aliasing model, this article simulates ten dual-signal aliasing models (Table 3). Each model generates 100 aliased signals with aliasing degrees of 25%, 50%, 75%, and 100% under the SNR of 0 dB, 4 dB, 8 dB, 12 dB, 16 dB, and 20 dB and then conducts recognition accuracy tests of the aliased signals under the fusion mode. The test results are shown in Figure 18. Figure 18a shows the results of the recognition rate test under multimodal features with 100% of the aliasing degree. The lowest % recognition rate of 98% is achieved with the EQFM + DQPSK, EQFM + 4FSK, EQFM + 32QAM, and EQFM + 8PSK models at the SNR of 0 dB. Figure 18b shows the results of the recognition rate test under multimodal features at 75% of aliasing, compared with the recognition results at 100% of aliasing; the recognition accuracy of the EQFM + DQPSK model and LFM + 2FSK model is improved by 1% when the SNR is 0 dB, and the recognition rate of the EQFM + 32QAM model is improved by 2%. Figure 18c,d show the results of the recognition rate test under multimodal features when the mixing degree is 50% and 25%, and the recognition rate of the ten two-signal mixing models can reach 100% when the SNR is higher than 0 dB under these two mixing degrees. An increase in the degree of aliasing leads to a decrease in the recognition rate. However, the TRMM method can still significantly improve the recognition accuracy of the two-signal aliasing models at a low SNR and high degrees of aliasing.

#### 5.3.3. Analysis of Three-Signal Aliasing Model Identification Results

In order to verify the recognition effect of the TRMM method in the three-signal time-frequency aliasing model, this article simulates the three-signal aliasing model of four kinds of signals (Table 3). Each model generates 100 aliasing signals with aliasing degrees of 25%, 50%, 75%, and 100%, respectively, under an SNR of 0 dB, 4 dB, 8 dB, 12 dB, 16 dB, and 20 dB to test the recognition accuracy of aliasing signals under the modal fusion mode. The test results are shown in Table 5. The EQFM + 16QAM + 32QAM model has the lowest % recognition rate of 98% when the SNR is 0 dB and the aliasing degree is 100%. With the decrease in the aliasing degree and the increase in the SNR, the recognition rate of the model increases. When the SNR is 0 dB, and the aliasing degree is 50%, the recognition rate of the model can reach 100%. When the SNR is higher than 0 dB, the recognition rate of the proposed model is 100% under different aliasing degrees. The LFM + 2FSK + 4FSK model has a high recognition rate, and the recognition rate can reach 100% under different SNR and aliasing degrees. The recognition rate of the LFM + BPSK + 8PSK model is 99% when the SNR is 0 dB, the aliasing degree is 100% and 75%, and the recognition rate increases to 100% when the SNR is 0 dB. The aliasing degree is 50% and 25%. When the SNR is higher than 0 dB, the recognition rate of the proposed model is 100% under different aliasing degrees. The recognition rate of the EQFM + BPSK + DQPSK model is 98% when the SNR is 0 dB and the aliasing degree is 100%. When the SNR is 0 dB, and the aliasing degree is 50%, the recognition rate is increased to 100%. When the SNR is higher than 0 dB, the recognition rate of the proposed model is 100% under different aliasing degrees. The experimental results show that the lowest recognition rate of the three-signal aliasing model can reach 98%, even when the SNR is 0 dB, and the aliasing degree is 100%.

#### 5.3.4. Analysis of Four-Signal Aliasing Model Identification Results

**Figure 19:**
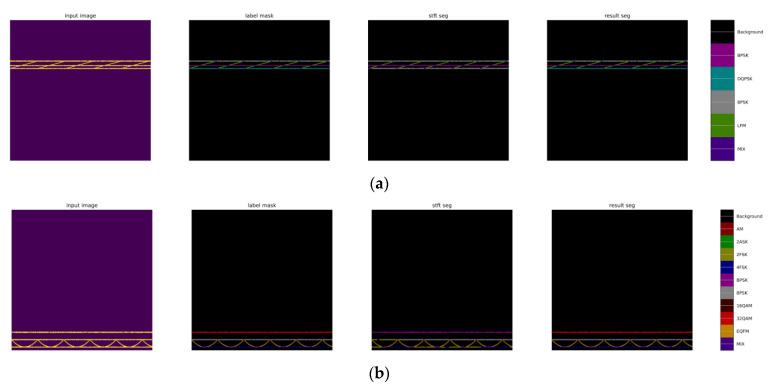
Examples of the recognition results of the TRMM method ((**a**): BPSK + DQPSK + 8PSK + EQFM; (**b**): 8PSK + 32QAM + 4FSK + EQFM).

In order to further verify the recognition effect of the TRMM method in the four-signal aliasing model, this article simulates six four-signal aliasing models (Table 3). Each model generates 100 aliased signals with aliasing degrees of 25%, 50%, 75%, and 100% under the SNR of 0 dB, 4 dB, 8 dB, 12 dB, 16 dB, and 20 dB, respectively. In the recognition results of the four-signal aliasing model, the TRMM effectively corrects the error of unimodal feature recognition, as exemplified by the BPSK + DQPSK + 2FSK + EQFM model. The 8PSK + 32QAM + 4FSK + EQFM model have been shown in Figure 19. In the figure, the input image picture is the time-frequency diagram of the aliased signal, the label mask picture is the label, the stft seg picture is the recognition result of the time-frequency diagram unimodal network, and the result seg picture is the recognition result of the fusion network.

In Figure 19a, the time-frequency unimodal network identifies the DQPSK signal (represented by the color cyan) as an 8PSK signal (represented by the color grey). In Figure 19b, the time-frequency unimodal network identifies the 32QAM signal (represented by the color red) as a BPSK signal (represented by the color pink). In the TRMM, after weighting the results of the identification of the time-frequency features according to the results of the identification of the wave-frequency features, the correct classification was achieved.

**Figure 20:**
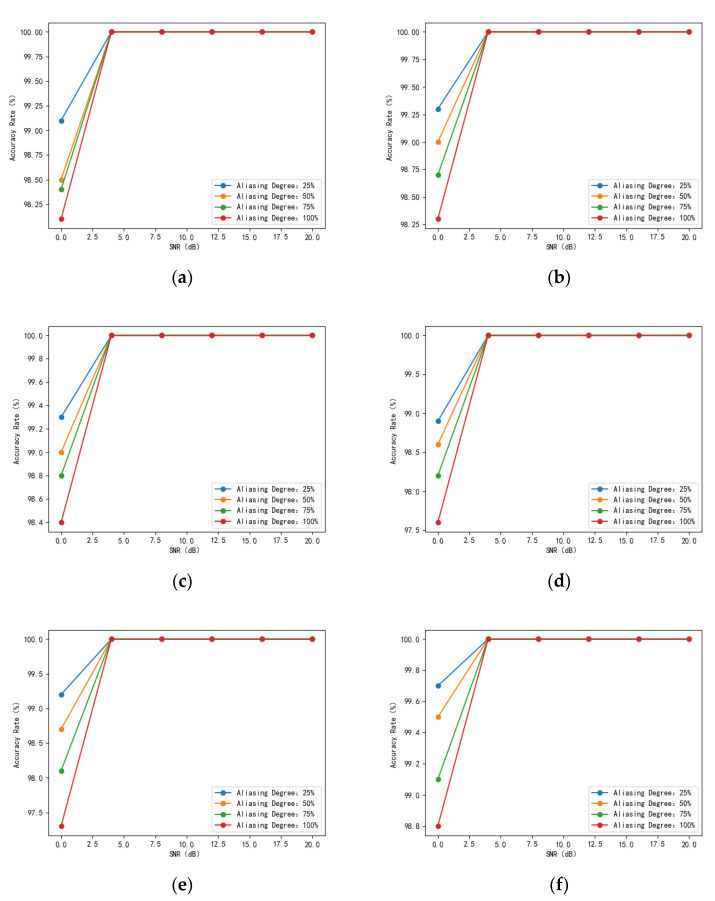
Comparison of recognition rates of four-signal aliasing models ((**a**). 8PSK + 32QAM + 4FSK + EQFM model recognition rate variation graph; (**b**). 16QAM + 2ASK + AM + LFM model recognition rate variation graph; (**c**). 16QAM + 4FSK + AM + LFM model recognition rate variation graph; (**d**). 16QAM + 32QAM + 4FSK + EQFM model recognition rate variation graph; (**e**). BPSK+ DQPSK + 8PSK + LFM model recognition rate variation graph; (**f**). BPSK + 4FSK + 2FSK + EQFM model recognition rate variation graph).

The test results of the four-signal aliasing model in the test set under different degrees of aliasing are statistically shown in Figure 20. It can be seen that the recognition rate of the aliased signals decreases with the increase in the degree of aliasing. The Figure 20e model has the lowest recognition rate at an SNR of 0 dB and a 100% aliasing degree, with a recognition rate of 97.3%. When the SNR is greater than 4 dB, the recognition rate of all four-signal aliasing models can reach 100%.

#### 5.3.5. Comparison of Recognition Performance of Different Algorithms

In order to further verify the performance of the TRMM method in dual-signal model recognition, Table 6 compares the recognition performance of the dual-signal aliasing model at an SNR of 0 dB. Table 6 shows that the recognition rates of Refs. [13,20] show a decreasing trend with a significant increase in the degree of blending. When $M_{f}>50\%$, the recognition rate decreases sharply. This is because the signal’s length constrains the loop accumulation estimation in [20], and Ref. [13] trains the DCNN network with one modal feature of a single signal. The results are directly output by the network without any optimization. The DCNN network is suitable for processing data with a spatial structure, while U-Net is a network architecture designed for image segmentation tasks. In the processing of feature maps, U-Net is obviously more suitable. The recognition rate of the TRMM method is less affected by the degree of aliasing, and the average recognition rate still reaches 99% when $M_{f}=100\%$ and the SNR is 0dB.

In order to further validate the performance of the TRMM method in the intra-class recognition of the three-signal mashup model, a comparison of the recognition performance of the three-signal mashup model at an SNR of 0 dB is given in Table 7. Ref. [21] maps the features to a high-dimensional space. It seeks the optimal classification hyperplane using a support vector machine to achieve signal recognition, but it usually applies to small sample datasets. Table 7 shows that the TRMM method still maintains a high recognition rate in intra-class signal recognition for the three-signal aliasing model.

Table 8 compares the intra-class signal recognition performance of the four-signal aliasing models at an SNR of 0 dB. Ref. [14] uses the Seg-Net network to extract the time-frequency map features. Although Seg-Net is also a network architecture for image segmentation tasks, its encoder–decoder structure does not have skip connection layers and cannot capture features at different scales. The algorithm proposed in this chapter not only uses the U-Net network to capture and fuse multi-scale features, but also selects the time-frequency map and wave-frequency map as the feature input, which enhances the network’s understanding of signal features and further improves the segmentation accuracy. Compared with other methods, the method in this article still has a high recognition rate after adding the signal aliasing model.

## 6. Conclusions

Addressing the issue of the weak representation capabilities of unimodal features in time-frequency graphs, which hinders the full exploitation of homogeneous or heterogeneous data features and leads to a low recognition rate of intra-class signals, this article proposes the TRMM method. The TRMM method introduces wave-frequency graphs into signal features and utilizes multimodal feature fusion to identify potential correlations among multimodal features, maintain correlation constraints, significantly enhance the learning capability and generalization ability of the network, and effectively distinguish eleven types of single signals and twenty types of mixed signals. In summary, the main contributions of the TRMM method lie in its innovative multimodal feature fusion technology and the introduction of wave-frequency graph features, which significantly improve the recognition accuracy of the time-frequency mixed signals. The simulation results show that the proposed method has a better classification ability than other unimodal networks; at an SNR of 0 dB and a mixing degree of 100%, the average recognition accuracy of the time-frequency mixed signals can reach at least 98%. However, further research is needed to improve the recognition rate for signals with different powers and time-frequency mixed signals.

## Figures and Tables

**Table 1:** Unimodal recognition rate statistics.

Type of Signal	Recognition Accuracy of Time-Frequency Diagrams	Recognition Accuracy of Wave-Frequency Diagrams
AM	1	1
2ASK	1	1
2FSK	0.71	0.56
4FSK	0.94	0.77
BPSK	1	1
DQPSK	0.16	0.77
8PSK	0.87	1
16QAM	0.82	0.44
32QAM	0.45	0.97
LFM	1	0.85
EQFM	1	0.86

**Table 2:** Weights for signal fusion weighting.

Type of Signal	Weights for Weighting Time-frequency Diagrams	Weights for Weighting Wave-Frequency Diagrams
AM	0.5	0.5
2ASK	0.5	0.5
2FSK	0.7	0.3
4FSK	0.9	0.1
BPSK	0.5	0.5
DQPSK	0.1	0.9
8PSK	0.45	0.55
16QAM	0.9	0.1
32QAM	0.05	0.95
LFM	0.9	0.1
EQFM	0.9	0.1

**Table 3:** Modeling of various types of mixed signals.

Category of Data	Overlapping Patterns	Collection of Signals
Training set	Single-signal	AM, 2ASK, 2FSK, 4FSK, BPSK, DQPSK, 8PSK, 16QAM, 32QAM, LFM, EQFM
	Dual-signal	LFM + AM, LFM + 2ASK, LFM + 2FSK, LFM + 4FSK, LFM + BPSK, LFM + DQPSK, LFM + 8PSK, LFM + 16QAM, LFM + 32QAM, EQFM + AM, EQFM + 2ASK, EQFM + 2FSK, EQFM + 4FSK, EQFM + BPSK, EQFM + DQPSK, EQFM + 8PSK, EQFM + 16QAM, EQFM + 32QAM
	Three-signal	EQFM + 16QAM + 32QAM, EQFM + 16QAM + DQPSK, LFM + 2FSK + 4FSK, LFM + BPSK + 8PSK, LFM + AM + 2ASK
Test set	Single-signal	AM, 2ASK, 2FSK, 4FSK, BPSK, DQPSK, 8PSK, 16QAM, 32QAM, LFM, EQFM
	Dual-signal	LFM + AM, EQFM + 2ASK, LFM + 2FSK, EQFM + 4FSK, LFM + BPSK, EQFM + DQPSK, EQFM + 8PSK, LFM + 16QAM, EQFM + 32QAM, EQFM + LFM
	Three-signal	EQFM + 16QAM + 32QAM, LFM + 2FSK + 4FSK, LFM + BPSK + 8PSK, EQFM + BPSK + DQPSK
	Four-signal	EQFM + 4FSK + 8PSK + 32QAM, LFM + AM + 4FSK + 16QAM, BPSK + 2ASK + 8PSK + LFM, BPSK + DQPSK + 2FSK + EQFM, EQFM + 16QAM + 32QAM + 4FSK, 16QAM + 2ASK + AM + LFM

**Table 4:** Variation in recognition rate with SNR for single signal in time-frequency plot mode and multimodal mode.

SNR	0 dB		4 dB		8 dB		12 dB	
Recognition Mode	SMF	TRMM	SMF	TRMM	SMF	TRMM	SMF	TRMM
BPSK	100%	100%	100%	100%	100%	100%	100%	100%
8PSK	87.4%	100%	92.6%	100%	99.3%	100%	100%	100%
2FSK	71.1%	99.3%	83.4%	100%	97.8%	100%	100%	100%
4FSK	94.9%	100%	100%	100%	100%	100%	100%	100%
16QAM	82.7%	100%	96.0%	100%	99.1%	100%	100%	100%
32QAM	45.7%	100%	88.7%	100%	97.4%	100%	100%	100%
2ASK	100%	100%	100%	100%	100%	100%	100%	100%
AM	100%	100%	100%	100%	100%	100%	100%	100%
DQPSK	16.1%	99.1%	78.9%	100%	93.7%	100%	100%	100%
EQFM	100%	100%	100%	100%	100%	100%	100%	100%
LFM	100%	100%	100%	100%	100%	100%	100%	100%

**Table 5:** Variation in recognition rate of the three-signal aliasing model under different aliasing degrees with the SNR.

$M_{f}$	25%		50%		75%		100%	
SNR	0 dB	4 dB	0 dB	4 dB	0 dB	4 dB	0 dB	4 dB
EQFM + 16QAM + 32QAM	100%	100%	100%	100%	99%	100%	98%	100%
LFM + 2FSK + 4FSK	100%	100%	100%	100%	100%	100%	100%	100%
LFM + BPSK + 8PSK	100%	100%	100%	100%	99%	100%	99%	100%
EQFM + BPSK + DQPSK	100%	100%	100%	100%	100%	100%	99%	100%

**Table 6:** The average recognition rate of different algorithms for dual-signal aliasing model under different aliasing degrees.

Algorithm Name	$M_{f}=25\%$	$M_{f}=50\%$	$M_{f}=75\%$	$M_{f}=100\%$
Ref. [20]	98.56%	93.41%	28.28%	14.55%
Ref. [13]	96.17%	71.17%	57.33%	47.17%
TRMM	100%	100%	99.7%	99.1%

**Table 7:** Average recognition rates of different algorithms for the three-signal overlapping model with different degrees of overlapping.

Algorithm Name	$M_{f}=25\%$	$M_{f}=50\%$	$M_{f}=75\%$	$M_{f}=100\%$
Ref. [21]	95.48%	93.375%	92.35%	92.17%
TRMM	100%	100%	99%	99.5%

**Table 8:** Average recognition rates of different algorithms for the four-signal aliasing model at different aliasing degrees.

Algorithm Name	$M_{f}=25\%$	$M_{f}=50\%$	$M_{f}=75\%$	$M_{f}=100\%$
Ref. [13]	92%			
Ref. [14]	98.97%	98.8%	98.13%	98.01%
TRMM	99.25%	98.88%	98.55%	98.08%

## Data Availability

Data are contained within this article.

## References

[1] Raviteja P., Phan K.T., Hong Y., Viterbo E. Orthogonal Time Frequency Space (OTFS) Modulation Based Radar System. Proceedings of the 2019 IEEE Radar Conference (RadarConf).

[2] Hinton G.E., Osindero S., Teh Y.W. (2006). A Fast Learning Algorithm for Deep Belief Nets. Neural Comput..

[3] Krizhevsky A., Sutskever I., Hinton G.E. (2012). ImageNet classification with deep convolutional neural networks. Commun. ACM.

[4] O’Shea T.J., Corgan J., Clancy T.C. Convolutional radio modulation recognition networks. Proceedings of the International Conference on Engineering Applications of Neural Networks.

[5] West N.E., O’Shea T. Deep Architectures for Modulation Recognition. Proceedings of the 2017 IEEE International Symposium on Dynamic Spectrum Access Networks (DySPAN).

[6] Pang J. (2023). Research on Communication Signal Modulation Recognition Technology Based on DAE_Transformer.

[7] Wang H., Zhang R., Huang Y. (2024). Spread Spectrum and Conventional Modulation Signal Recognition Method Based on Generative Adversarial Network and Multi-modal Attention Mechanism. J. Electron. Inf. Technol..

[8] Kögel M., Brand S., Altmann F. Machine Learning Based Data and Signal Analysis Methods for Application in Failure Analysis (2022 Update). Proceedings of the International Symposium for Testing and Failure Analysis.

[9] Wang Y., Wang J., Zhang W., Yang J., Gui G. (2020). Deep Learning-based Cooperative Automatic Modulation Classification Method for MIMO Systems. IEEE Trans. Veh. Technol..

[10] Yu S. (2021). Research on Modulation Recognition of Non Cooperative Communication Systems Based on Artificial Neural Networks.

[11] Xu Y. (2020). Research on Modulation Recognition of Digital Signals Based on Multi Feature Extraction.

[12] Li J.C. (2023). Recognition of Time-Frequency Aliasing Modulation Signals Based on Lightweight Networks.

[13] Liu Z., Li L., Xu H., Li H. A Method for Recognition and Classification for Hybrid Signals Based on Deep Convolutional Neural Network. Proceedings of the International Conference on Electronics Technology.

[14] Pan N. (2022). Identification and Separation of Time-Frequency Aliasing Signals in Complex Electromagnetic Environments.

[15] Wang H.F. (2023). Sentiment Analysis Based on Multimodal Feature Fusion.

[16] Otsu N. (2007). A Threshold Selection Method from Gray-Level Histograms. IEEE Trans. Syst. Man Cybern..

[17] Zilberman E.R., Pace P.E. Autonomous Time-Frequency Morphological Feature Extraction Algorithm for LPI Radar Modulation Classification. Proceedings of the IEEE International Conference on Image Processing.

[18] Dai H.J. (2023). Research on image segmentation method based on improved UNet. Inf. Technol. Inf..

[19] Lin Q.H. (2022). Research on Modulation Recognition Technology and Influence Factor of Digital Communication.

[20] Yang Z., Hua P. (2016). Modulation Recognition for Mixed Signals in Single Channel. J. Univ. Inf. Eng..

[21] Li P.B. (2020). Research on the Modulation Recognition Algorithm for Single-Channel Time-Frequency Aliasing Signals.

